# Re-irradiation for local primary-recurrence esophageal squamous cell carcinoma treated with IMRT/VMAT

**DOI:** 10.1186/s13014-023-02265-w

**Published:** 2023-07-10

**Authors:** Geng Xiang, Chunsheng Xu, Guangjin Chai, Bo Lyu, Zhaohui Li, Bin Wang, Mei Shi, Lina Zhao

**Affiliations:** 1 Department of Radiation Oncology, Xijing Hospital, Air Force Medical University, Xi’an, 710032 Shaanxi China; 2 Department of Gastrointestinal Surgery, Xijing Hospital, Air Force Medical University, Xi’an, 710032 Shaanxi China

**Keywords:** Esophageal squamous cell carcinoma, Local primary-recurrence, Re-irradiation, Intensity modulated radiation therapy, Volumetric modulated arc therapy, Overall survival, After recurrence survival

## Abstract

**Purpose:**

Local primary-recurrence of esophageal squamous cell carcinoma (ESCC) after definitive treatment has the potential for increasing overall survival with re-irradiation (Re-RT), especially with advanced technique. This study aimed to evaluate the efficacy and toxicities of Re-RT using intensity-modulated radiotherapy (IMRT)/volumetric modulated arc therapy (VMAT) for local primary-recurrence of ESCC.

**Materials and methods:**

A total of 130 ESCC patients with local primary-recurrence from Xijing hospital between 2008 and 2021 were enrolled and 30 patients underwent IMRT/VMAT based salvage Re-RT. Cox regression analysis was used to analyze the prognostic factors for overall survival (OS) and after recurrence survival (ARS). The toxicities of 30 patients receiving Re-RT were also assessed.

**Results:**

The median OS and ARS of the 130 recurrent patients were 21 months (1−164 months) and 6 months (1−142 months). The 1-, 2-, and 3-year OS rates were 81.5%, 39.2%, and 23.8%, respectively. Besides, the 1-, 2-, and 3-year ARS rates were 30.0%, 10%, and 6.2%. Multivariate analysis showed that Re-RT ± chemotherapy (*p =* 0.043) and chemotherapy alone (*p* < 0.001) and esophageal stents (*p =* 0.004) were independent prognostic factors for OS. The median OS of 30 patients treated with Re-RT were significantly better than that of 29 patients treated with chemotherapy (34.5 months vs. 22 months, *p =* 0.030). Among 30 ESCC patients treated with Re-RT, the median OS and ARS were 34.5 months (range 12–163 months) and 6 months (range 1−132 months), respectively. The recurrence-free interval (RFI) (> 12 months) and initial radiation dose (> 60 Gy) were significantly associated with improved OS. Radiation esophagitis (Grade 1–2) occurred in 16 patients and myelosuppression (Grade1−2) occurred in 10 patients. Grade 3 toxicities (radiation esophagitis and myelosuppression) were only 13.3%. There were no grade 4 toxicities.

**Conclusion:**

Our results demonstrated that IMRT/VMAT-based Re-RT was an effective therapeutic option for ESCC patients with local primary-recurrence compared with chemotherapy alone or without any treatment. Re-RT had improved OS but unfavorable ARS.

**Supplementary Information:**

The online version contains supplementary material available at 10.1186/s13014-023-02265-w.

## Introduction

Esophageal cancer (EC) is the sixth leading cause of cancer death worldwide because of its poor prognosis, with a 5-year survival of only 20% [1, 2]. Esophageal squamous cell carcinoma (ESCC) is known as the predominant histologic subtype of EC worldwide, characterized by a high recurrence and mortality rate [3]. Definitive chemoradiotherapy (dCRT) is the standard therapy for unresectable locally advanced ESCC [4]. However, local recurrence (LR) after dCRT was still the main failure pattern (~ 50%) [5, 6]. Once recurrence occurs, the survival rate at five-years drops to 0–11% [7]. It is of great importance to balance disease control and toxicities when considering salvage treatments.

Although salvage surgery has curative potential, high rates of mortality, anastomotic leak, and pulmonary complications limit the number of patients who are candidates for salvage surgery [8–10]. It is reported that chemotherapy, radiotherapy (RT) or the combined methods could provide survival benefits for salvage treatment of recurrent esophageal cancer (REC), but there is still no consensus [11, 12]. Chemotherapy alone is preferred as a systemic treatment for patients with metastatic disease or multiple-site recurrence [12, 13]. However, salvage systemic chemotherapy after LR was not very satisfactory, with an estimated median overall survival (OS) duration of only 5 months [14]. It is worth noting that Re-RT has been reported to have favorable clinical outcomes for patients with recurrent head and neck tumors, lung cancer, and rectal cancer [15–17]. There is little doubt about the palliative effect of Re-RT in the management of REC. Several studies have reported improvement of symptoms with an objective response rate of 55–91% [18, 19]. Usually, Re-RT is used with caution because of poor blood supply, less tumor sensitivity and the increased complications such as esophageal perforation or stenosis [7, 19, 20]. Re-RT has been reported to achieve long-term survival in carefully selected REC patients treated with three-dimensional conformal radiotherapy (3D-CRT) or intensity-modulated radiotherapy (IMRT) technique [13]. In the past decades, modern RT techniques including IMRT and volumetric modulated arc therapy (VMAT) enable high dose to limited volumes, excluding critical normal tissues, and therefore increase the safety of Re-RT [21]. A few studies have indicated that IMRT/VMAT based Re-RT was superior to conventional RT technique in terms of efficacy and toxicities in REC patients, but the number of enrolled patients was relatively small and no study reported IMRT/VMAT only based Re-RT [22, 23]. To the best of our knowledge, there is no published report to date which analyzes the efficacy and toxicity of initial RT and Re-RT/re-chemoradiotherapy (Re-CRT) for ESCC patients treated with IMRT/VMAT only.

In the present study, we retrospectively analyzed the clinical outcomes, toxicities and prognostic factors of IMRT/VMAT based Re-RT/Re-CRT for ESCC patients with local primary-recurrence.

## Methods

### Patients

In the present study, we retrospectively evaluated 130 ESCC patients with local primary-recurrence who were admitted to Xijing Hospital between April 2008 and March 2021. All patients were treated with definitive RT/dCRT as the initial treatment. All eligible patients with Re-RT met the following criteria: (1) local primary-recurrence (recurrence in primary tumor site) diagnosed by histology or imaging examination without regional lymph node recurrence and distant metastasis; (2) a history of initial definitive radiation receiving a radiation dose > 50 Gy and elective nodal irradiation; (3) (Zubrod/ECOG/WHO [ZPS]) 1–2; (4) IMRT/VMAT was applied in both initial and Re-RT treatment; (5) recurrence was in-field or marginal. In-field and marginal recurrence were defined as ≥ 95% and 50%−94% overlapping volume of initial planning target volume and the recurrent tumor, respectively. This study was approved by the Ethics Committee of the First Affiliated Hospital of Air Force Medical University (ethical approval number: KY20172035−2).

### Follow-up

Patients were re-evaluated for disease control, complications, and survival 1 month after treatment completion, then were followed up every 3 months in the first year, every 6 months from the second to the fifth year, and annually thereafter. The endpoint was OS, which was calculated from the date of diagnosis to death or last follow-up. The after recurrence survival (ARS) time was defined as the time of interval from the date of relapse to the date of death or last follow-up. The recurrence-free interval (RFI) was defined as the time of interval from the end of initial treatment to the recurrence diagnosis. Toxicity was assessed according to the National Cancer Institute Common Terminology Criteria for Adverse Events version 4.0 (CTCAE v4.0).

#### Statistical analysis

All statistical tests for data analysis were performed using SPSS version 22.0 (IBM Corporation, Armonk, NY, USA). Survival curves were estimated by use of the Kaplan-Meier method and groups were compared for their survival rates by the log-rank test. Both univariate and multivariate analysis were performed by use of Cox regression models to identify significant prognostic factors. Hazard ratios (HRs) and 95% confidence intervals (CIs) were estimated for each prognostic factor. A *P* value of < 0.05 was considered to be statistically significant.

## Results

### Patients and treatment

The baseline characteristics of 130 ESCC patients with local primary-recurrence were summarized in Table 1. The median age was 67 years (range 39–87 years). 96 patients (73.8%) were males and 111 patients (85.4%) had T3 and T4 disease. 96 patients (73.8%) showed stage III/IV at initial presentation. The median tumor length of these lesions at initial diagnosis was 5 cm (range, 2–30 cm). The median initial radiation dose was 60 Gy (range 50–70 Gy), and 92 patients (70.8%) were initially treated with concurrent chemotherapy. The salvage treatment included Re-RT with/without chemotherapy (n = 30), chemotherapy alone (n = 29), esophageal stents (n = 8), observation (n = 58) and other treatments (n = 5). Actually, of the 58 patients with observation, 45 patients (77.6%) received “best supportive care”, and 13 patients (22.4%) refused treatment due to family economic factors, worries about the side effects, etc.

**Table 1:** Characteristics of 130 ESCC patients with local primary-recurrence at initial treatment

Variable	Total (%) (n = 130)	Re-RT (n = 30)	Chemotherapy (n = 29)	Esophageal stent (n = 8)	Other treatment (n = 5)	Without treatment (n = 58)
Gender						
Male	96(73.8)	24(18.5)	22(16.9)	5(3.8)	5(3.8)	40(30.8)
Female	34(26.2)	6(4.6)	7(5.4)	3(2.3)	0(0.0)	18(13.8)
Age						
≤ 65	57(43.8)	13(10.0)	14(10.8)	3(2.3)	4(3.1)	23(17.7)
> 65	73(56.2)	17(13.1)	15(11.5)	5(3.8)	1(0.8)	35(26.9)
PS						
0/1	82(63.1)	24(18.5)	18(13.8)	5(3.8)	3(2.3)	32(24.6)
2	48(36.9)	6(4.6)	11(8.5)	3(2.3)	2(1.5)	26(20.0)
T stage						
1/2	19(14.6)	7(5.4)	1(0.8)	0(0.0)	0(0.0)	11(8.5)
3/4	111(85.4)	23(17.7)	28(21.5)	8(6.2)	5(3.8)	47(36.2)
N stage						
0/1	107(82.3)	28(21.5)	23(17.7)	7(5.4)	4(3.1)	45(34.6)
2/3	23(17.7)	2(1.5)	6(4.6)	1(0.8)	1(0.8)	13(10.0)
Clinical stage						
I/II	34(26.2)	9(6.9)	7(5.4)	3(2.3)	0(0.0)	15(11.5)
III/IV	96(73.8)	21(16.2)	22(16.9)	5(3.8)	5(3.8)	43(33.1)
Tumor location						
Upper and middle thoracic	80(61.5)	17(13.1)	19(14.6)	5(3.8)	4(3.1)	35(26.9)
Lower thoracic	50(38.5)	13(10.0)	11(8.5)	3(2.3)	1(0.8)	23(17.7)
Tumor length(cm)						
≤ 5	66(50.8)	18(13.8)	12(9.2)	5(3.8)	2(1.5)	29(22.3)
> 5	64(49.2)	12(9.2)	17(13.1)	3(2.3)	3(2.3)	29(22.3)
Radiotherapy technique						
IMRT	51(39.2)	21(16.2)	7(5.4)	3(2.3)	0(0.0)	20(15.4)
VMAT	79(60.8)	9(6.9)	22(16.9)	5(3.8)	5(3.8)	38(29.2)
Initial radiation dose (Gy)						
≤ 60	75(57.7)	13(10.0)	20(15.4)	4(3.1)	3(2.3)	35(26.9)
> 60	55(42.3)	17(13.1)	9(6.9)	4(3.1)	2(1.5)	23(17.7)
Concurrent chemotherapy						
Yes	92(70.8)	22(16.9)	22(16.9)	4(3.1)	5(3.8)	39(30.0)
No	38(29.2)	8(6.2)	7(5.4)	4(3.1)	0(0.0)	19(14.6)
RFI (months)						
≤ 12	80(61.5)	9(6.9)	19(14.6)	6(4.6)	2(1.5)	44(33.8)
> 12	50(38.5)	21(16.2)	10(7.7)	2(1.5)	3(2.3)	14(10.8)

The patients and treatment characteristics of 30 ESCC patients with Re-RT were summarized in Table S1. The median age was 68.5 years (range 44–84 years), 24 (80.0%) were males and 6 (20.0%) were females. Considering PS at recurrence diagnosis, 19 (63.3%) of patients were 2. 24 patients (80.0%) had T3 and T4 disease, 19 (63.3%) had lymph node metastasis, and 17 (56.7%) had stage III/IV at the initial diagnosis. The tumor location was the upper and middle thoracic esophagus in 15 (50.0%) patients and the lower thoracic esophagus in 15 (50.0%) patients. The median tumor length was 5 cm (range, 2–19 cm) at initial treatment and 7.5 cm (range, 5–13 cm) at recurrence. 24 patients (80.0%) and 6 patients (20.0%) had in field and marginal recurrence, respectively. The median RFI after initial radiotherapy for Re-RT patients was 18 months (6−118 months).

For initial radiation treatment, 30 ESCC patients received IMRT (n = 21)/VMAT (n = 9) (1.8−2.0 Gy/fraction, 5 days/week). The initial dose range was 50.4–66.0 Gy, with a median dose of 61.6 Gy. Among the 22 cases initially treated with concurrent chemotherapy (median of 2 cycles, range 1–4 cycles), 10 (45.5%) received cisplatin and tegafur-gimeracil-oteracil potassium (S−1), 7 (31.8%) received cisplatin and fluorouracil (PF), whereas the remaining 5 (22.7%) received S−1 or others. For Re-RT treatment, the median dose was 55.5 Gy (25.3–63 Gy/1.8−2.0 Gy). IMRT (n = 10)/VMAT (n = 20) were applied for Re-RT. 4 patients received doses < 40 Gy was interrupted due to rapid progression. Detail dose limitations for normal organs were shown in Table S2. 13 patients (43.3%) received Re-CRT (median of 2 cycles chemotherapy, range 1–3 cycles), 5 received paclitaxel and cisplatin (TP), 2 received cisplatin combined with S−1, 2 received S−1 only, and 3 received capecitabine. The remaining 17 (56.7%) patients received Re-RT alone. The median total radiation dose was 115.4 Gy (range 86.9−126.6 Gy).

### Survival outcomes and prognostic factors for all recurrent patients

**Fig. 1:**
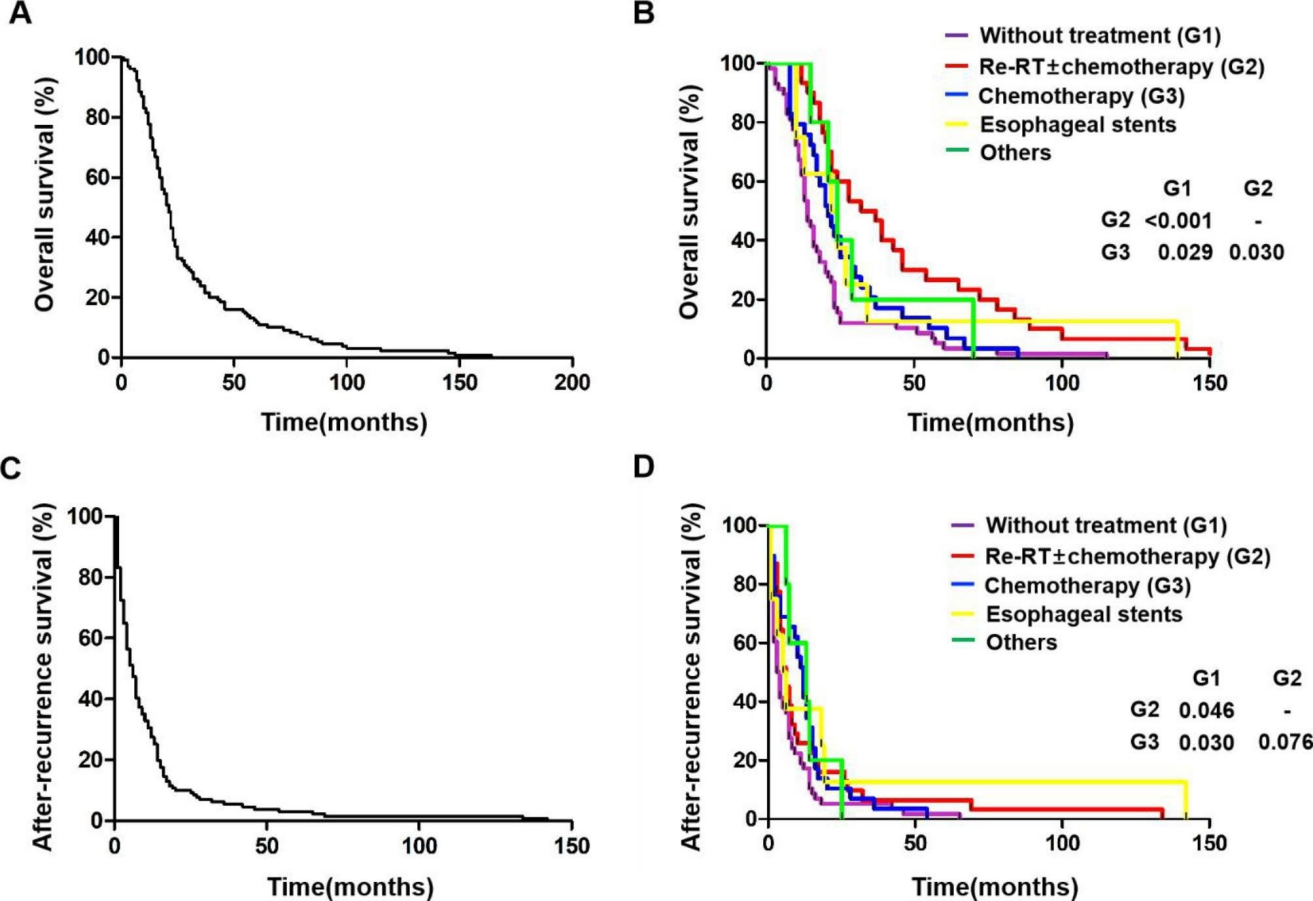
Kaplan-Meier survival curves. (A) Kaplan-Meier curve of OS for 130 ESCC patients with local primary-recurrence. (B) OS of 130 patients who received different salvage treatment. (C) Kaplan-Meier curve of ARS for 130 ESCC patients with local primary-recurrence. (B) ARS of 130 patients who received different salvage treatment

The median OS and ARS of the 130 patients were 21 months (1−164 months) (Fig. 1A) and 6 months (1−142 months) (Fig. 1C). The median OS and ARS of 30 patients with Re-RT (OS: 34.5 months vs. 14 months, *p* < 0.001; ARS: 6 months vs. 3 months, *p =* 0.046) and 29 patients with chemotherapy (OS: 22 months vs. 14 months, *p* = 0.029; ARS: 12 months vs. 3 months, *p =* 0.030) were significantly better than that of 58 patients without treatment (Fig. 1B and Fig. 1D). Compared to patients in the chemotherapy group, those in Re-RT had a better higher OS (*p =* 0.030), but no higher ARS (*p =* 0.076). Multivariate Cox regression analysis showed that PS score (*p =* 0.008), initial radiation dose (*p =* 0.039), RFI (*p* < 0.001), Re-RT ± chemotherapy (*p =* 0.043), chemotherapy alone (*p* < 0.001) and esophageal stents (*p =* 0.004) were independent prognostic factors associated with OS (Table S3). However, Re-RT ± chemotherapy, chemotherapy alone and esophageal stents were not independent prognostic factors associated with ARS (Figure S1 and Table S3).

### Survival outcomes and prognostic factors for 30 patients receiving Re-RT

**Fig. 2:**
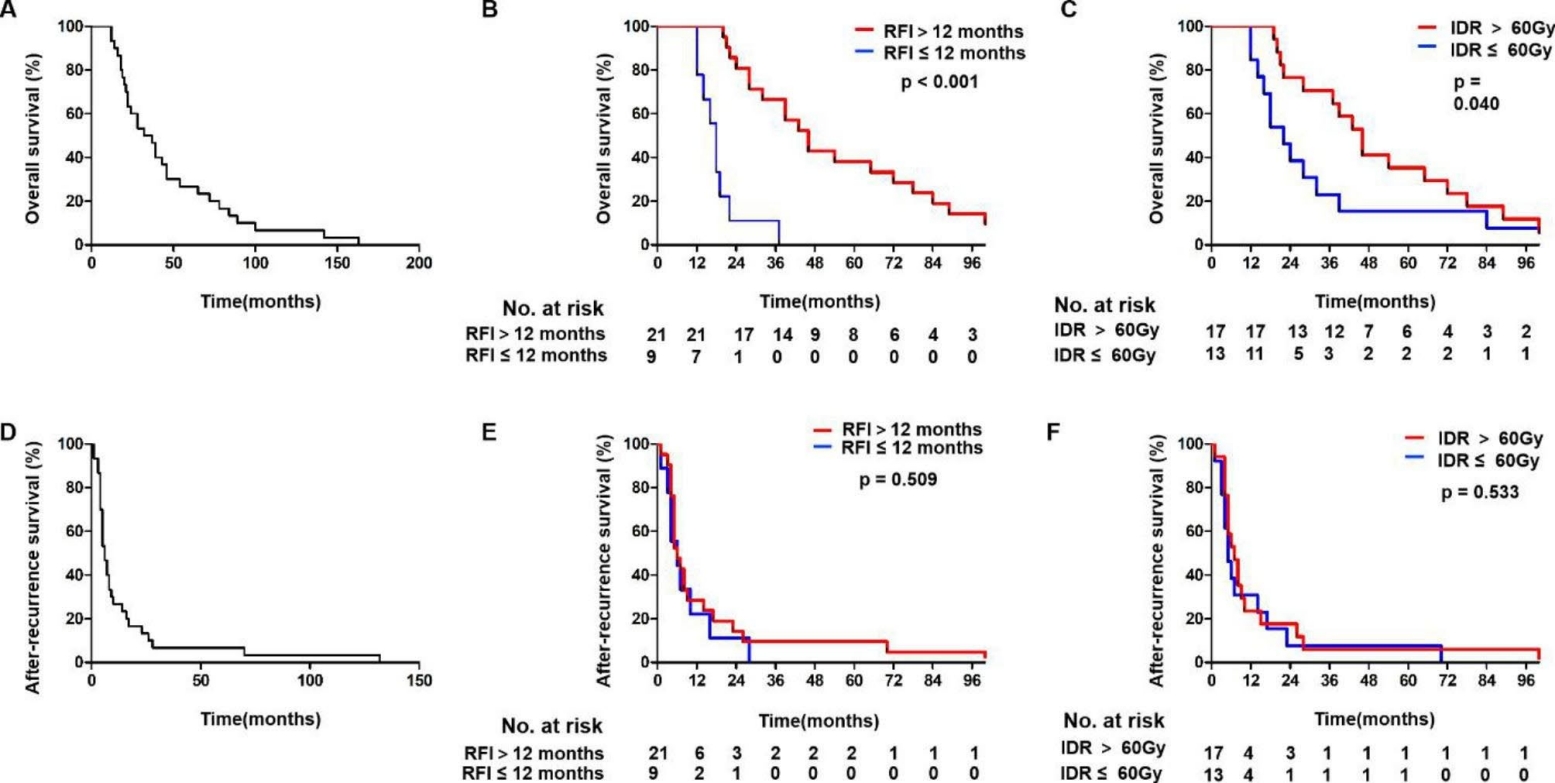
Kaplan-Meier survival curves. (A, D) Kaplan-Meier curve of OS and ARS for 30 ESCC patients receiving Re-RT with local primary-recurrence. (B, E) Kaplan-Meier curve of for 30 ESCC patients who had an RFI ≤ 12 months versus RFI > 12 months. (C, F) Kaplan-Meier curve of for 30 ESCC patients who received initial RT dose > 60 Gy versus ≤ 60 Gy

Of the 30 patients who received Re-RT, the median OS was 34.5 months (range 12–163 months), and 1-, 2-, and 3-years OS rates were 100.0%, 63.3% and 50.0%, respectively (Fig. 2A). The median ARS for the 30 patients with Re-RT was 6 months (range 1−132 months) and 1-, 2-, and 3-years ARS rates were 26.6%, 13.3%, and 6.7%, respectively (Fig. 2D). Details regarding the reasons for death are provided in Table S4. The most common reasons for death were metastasis or uncontrolled disease (56.7%), dysphagia (20.0%), and gastrointestinal bleeding (10.0%). The local control rate after recurrence was 43.3% in current study.

The results of univariate analyses for OS and ARS are summarized in Table 2. Smoking, alcohol abuse, RFI (< 12 months), and initial radiation dose (< 60 Gy) were associated with worse OS (*p* = 0.040, and *p* < 0.001, respectively) by univariate analysis. However, no prognostic factor for ARS was observed. The RFI and initial radiation dose (> 60 Gy) were prognostic factors for OS (*p* < 0.001 and *p* = 0.040, respectively) by multivariate analysis (Table 3and Fig. 2). Figure S2 showed that initial radiation dose > 60 Gy was not associated with favorable RFI (*p* = 0.158).

**Table 2:** Cox univariate analysis of the ARS and OS for 30 ESCC patients treated with Re-RT

Variable	n(%)	OS		ARS	
		HR(95%CI)	*P*	HR(95%CI)	*P*
Gender		0.578(0.232–1.441)	0.240	1.959(0.765–5.011)	0.161
Male	24(80.0)				
Female	6(20.0)				
Age		1.124(0.501–2.518)	0.777	0.855(0.386–1.891)	0.698
≤ 65	10(33.3)				
> 65	20(66.7)				
Alcohol abuse		2.082(0.958–4.525)	0.064*	0.936(0.449–1.953)	0.861
Yes	13(43.3)				
No	17(56.7)				
Smoking		2.265(1.037–4.950)	0.040*	0.773(0.367–1.628)	0.498
Yes	13(43.3)				
No	17(56.7)				
PS		1.253(0.542–2.894)	0.598	1.057(0.464–2.406)	0.896
1	11(36.7)				
2	19(63.3)				
T stage^#^		1.112(0.448–2.759)	0.819	1.226(0.492–3.057)	0.661
1/2	6(20.0)				
3/4	24(80.0)				
N stage^#^		1.920(0.858–4.298)	0.112	1.040(0.480–2.251)	0.922
0	11(36.7)				
1/2	19(63.3)				
Clinical stage^#^		1.596(0.742–3.434)	0.232	1.362(0.648–2.863)	0.415
I/II	13(43.3)				
III/IV	17(56.7)				
Tumor location^#^		1.269(0.611–2.638)	0.523	1.137(0.525–2.462)	0.745
Upper and middle thoracic	15(50.0)				
Lower thoracic	15(50.0)				
Tumor length(cm)^#^		1.641(0.770–3.498)	0.199	1.239(0.589–2.606)	0.572
≤ 5	18(60.0)				
> 5	12(40.0)				
RFI (months)		0.102(0.036–0.289)	< 0.001*	0.774(0.349–1.717)	0.529
≤ 12	9(30.0)				
> 12	21(70.0)				
Re-RT field		1.232(0.493–3.081)	0.655	1.243(0.497–3.110)	0.642
In field	24(80.0)				
Marginal	6(20.0)				
Concurrent chemotherapy		1.660(0.773–3.563)	0.193	1.818(0.840–3.934)	0.129
Yes	13(43.3)				
No	17(56.7)				
Chemotherapy for both course treatment		1.504(0.689–3.281)	0.305	2.162(0.951–4.914)	0.066
Yes	10(33.3)				
No	20(66.7)				
Re-Radiotherapy technique		0.929(0.426–2.026)	0.853	1.490(0.680–3.268)	0.319
IMRT	10(33.3)				
VMAT	20(66.7)				
Initial radiation dose (Gy)		0.521(0.247–1.097)	0.086*	0.800(0.383–1.670)	0.552
≤ 60	13(43.3)				
> 60	17(56.7)				
Re-RT dose (Gy)		1.082(0.514–2.277)	0.835	0.846(0.398–1.799)	0.664
≤ 50	12(40.0)				
> 50	18(60.0)				
Total radiation dose (Gy)		0.757(0.364–1.575)	0.363	0.689(0.329–1.442)	0.322
≤ 115	15(50.0)				
> 115	15(50.0)				

* Univariate analysis was used to calculate the p value of variables, and then multivariate analysis was performed for variables with p < 0.1 to analyze independent risk factors

^#^ Variables at initial diagnosis

**Table 3:** Cox multivariate analysis of the ARS and OS for 30 ESCC patients treated with Re-RT

Variable	n(%)	OS		ARS	
		HR(95%CI)	*P*	HR(95%CI)	*P*
Alcohol abuse		-	-	-	-
Yes	13				
No	17				
Smoking		-	-	-	-
Yes	13				
No	17				
RFI (months)		0.080(0.027–0.239)	< 0.001*	-	**-**
≤ 12	9				
> 12	21				
Initial radiation dose (Gy)		0.419(0.191–0.920)	0.030*	-	-
≤ 60	13				
> 60	17				

*P values less than 0.05 are highlighted in bold

**Fig. 3:**
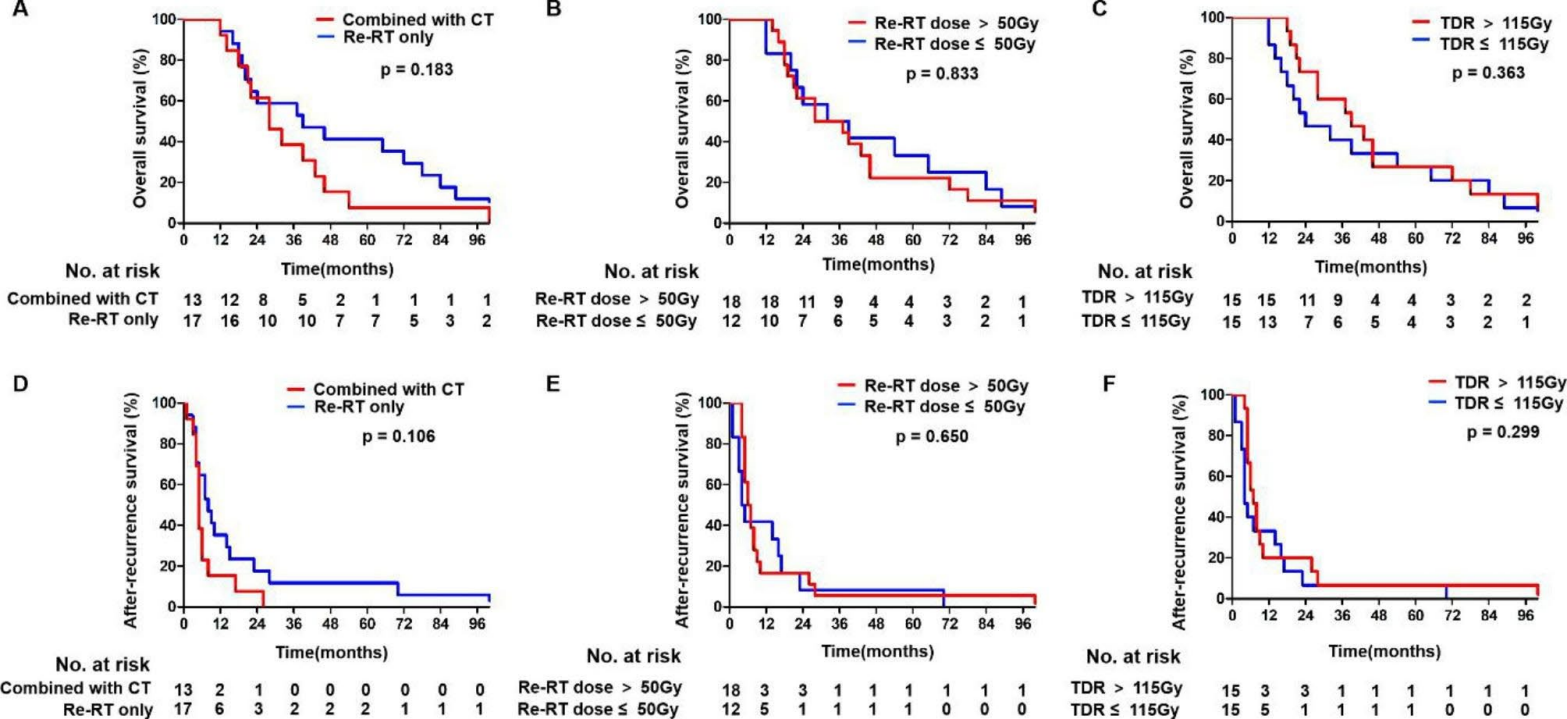
Kaplan-Meier survival curves. (A, D) Survival of patients who received Re-RT only versus Re-RT combined chemotherapy. (B, E) Survival of patients who received Re-RT dose > 50 Gy versus ≤ 50 Gy. (C, F) Survival of patients who received total RT dose > 115 Gy versus ≤ 115 Gy

The 30 patients were then stratified based on the use of concomitant chemotherapy, Re-RT dose, and total dose of radiation. Patients who received chemotherapy had the trend to have worse OS and ARS compared to those who received Re-RT alone, but there was no significant difference (*p* = 0.183 and *p* = 0.106, respectively, Fig. 3A and Fig. 3D). In addition, patients who received Re-RT dose > 50 Gy did not have a higher OS and ARS than patients who received Re-RT dose ≤ 50 Gy (*p* = 0.833 and *p* = 0.650, respectively, Fig. 3B and Fig. 3E). No statistical difference in OS and ARS was observed between the higher total radiation dose (> 115 Gy) and the lower total radiation dose (≤ 115 Gy) (*p* = 0.363 and *p* = 0.299, respectively, Fig. 3C and Fig. 3F).

### Toxicities for patients receiving Re-RT

Radiation esophagitis (RE) and myelosuppression are common acute toxicities (Table 4). RE occurred in 17 patients (grade 1–2 = 16 patients, grade 3 = 1 patient), and myelosuppression occurred in 13 patients (grade 1–2 = 10 patients, grade 3 = 3 patients). Grade 1–2 radiation pneumonitis, stenosis of the esophagus, and esophageal fistula occurred in 1 patient, 2 patients, and 4 patients, respectively. No esophageal perforation or radiation myelitis was observed in the study. No treatment-related deaths were recorded.

**Table 4:** Toxicities[n (%)]

Toxic Effects	Total(N = 30)	Grade 1–2	Grade 3
**Acute**			
Radiation esophagitis	17(56.7)	16(53.3)	1(3.3)
Myelosuppression	13(43.3)	10(33.3)	3(10.0)
Radiation pneumonitis	1(3.3)	1(3.3)	0(0)
Esophageal fistula	4(13.3)	4(13.3)	0(0)
**Late**			
Esophageal stricture	2(6.7)	2(6.7)	0(0)
Pericardial effusion	0(0)	0(0)	0(0)

## Discussion

LR occurs frequently after primary definitive RT or dCRT for ESCC. The prognosis of these patients is very poor, and most of these patients will die in 1 year without treatment [4, 20, 24]. However, therapeutic options remain limited, and no consensus regarding the optimal treatment has been reached. Re-RT for the management of recurrent ESCC has been reported to have beneficial effects on symptomatic control and curative potential [19, 20]. In the present study, the effectiveness of different salvage treatments were retrospectively analyzed, we found that PS score, initial radiation dose, RFI, Re-RT ± chemotherapy, chemotherapy alone and esophageal stents were independent prognostic factors associated with OS of 130 recurrent ESCC patients. However, there was no independent prognostic factor associated with ARS. For 30 recurrent ESCC patients receiving Re-RT, the RFI time > 12 months and initial radiation dose > 60 Gy were found to be independent prognostic factors for OS, but not ARS.

The role of salvage treatments in ESCC patients with LR in primary after RT is still controversial [21]. Previous studies suggested that Re-RT had been successfully used in several recurrent tumors except for ESCC with the development of RT techniques with encouraging outcomes [7]. There were several small size retrospective studies that reported the outcome for LR ESCC patients with Re-RT [12, 13, 23]. Chen reported that the survival rates for LR ESCC patients who received salvage radiotherapy and/or chemotherapy were comparable to those of patients who received surgery [20]. Katano described six patients who underwent Re-RT for locally recurrent EC patients following dCRT, with a median ARS of 13.6 months (range, 1.9–33.3 months) [25]. Hong also reported that Re-RT could improve the long-term survival of patients with LR ESCC, with a median survival time of 21 months and a 5-year OS of 13.08% [13]. Jingu reported that Re-RT for primary-recurrence in lymph nodes from esophageal cancer treated by definitive RT or by surgery with additional RT might be acceptable but unsatisfactory [22]. In our present study, we found that there was a significant increase in OS and ARS for patients who received Re-RT compared with the patients without treatment (*p* < 0.001 and *p* = 0.046). Compared to patients in the chemotherapy group, those in Re-RT had a better higher OS and no improved ARS (*p =* 0.030 and *p* = 0.076).

Previous studies showed LR was the most common failure pattern (57−71.3%), after dCRT [7, 26]. Besides LR was an independent prognostic factor for worse OS compared with regional lymph node relapse, which emphasized that control of the primary tumor plays a vital role in ESCC [13]. Therefore, the optimal radiation dose given for initial treatment was of great importance. In the present study, we focused on the 130 recurrent ESCC patients with local primary-recurrence and found that a dose > 60 Gy in the initial treatment was associated with better OS in univariate and multivariate analysis. We also found that initial radiation dose > 60 Gy was associated with longer OS but not ARS in 30 ESCC patients receiving Re-RT. The optimal dose of Re-RT for recurrent ESCC is hard to determine due to the need to balance the toxicities of normal tissues and organs and potential benefits [12]. Previous studies have shown that a radiation dose > 50 Gy showed better survival for recurrent ESCC, but the relatively higher dose did not yield significant improvement in the survival rates as well as toxicity [12, 20]. Wu reported that higher re-irradiation dose (55–60 Gy) can improve the long-term survival of patients with LR ESCC after RT, with tolerable toxicity [27]. Meanwhile, salvage radiation dose and total radiation dose did not affect OS (*p* = 0.835 and *p* = 0.363) and ARS (*p* = 0.664 and *p* = 0.322) in our present study in univariate analysis. Although we found that patients with RFI > 12 months had better OS (46 months vs. 18 months, *p* < 0.001) through univariate and multivariate analysis, patients receiving Re-RT with RFI > 12 months in ARS were similar to patients with RFI ≤ 12 months (6 months vs. 6months, *p* = 0.529).

It is well known that concurrent chemotherapy can improve the sensitivity of radiotherapy and improve the treatment effect [4]. However, there was a lack of clear evidence that Re-RT plus chemotherapy was beneficial for survival. Although Zhou [7] had reported that patients who received Re-RT plus chemotherapy had better ARS than those who did not, most cases of recurrent ESCC occurred in older patients, and CRT may not be optimal [13]. In the current study, only 43.3% of our patients received 1–2 courses of chemotherapy due to the poor physical condition, Re-RT combined with chemotherapy showed a worse trend in OS and ARS compared with Re-RT alone, even if the difference was not statistically significant (*p* = 0.193 and *p* = 0.129). Further studies are required to assess the effectiveness and toxicity of chemotherapy for Re-RT in highly selected patients and to tailor therapy to individuals to achieve the best possible outcomes.

Due to the special characteristics of esophagus, toxicity and quality of life play major roles in the evaluation the role of Re-RT for EC. The most important toxicities are dysphagia and consequent malnutrition. It was reported that approximately 20–25% of patients treated with chemoradiotherapy need parenteral nutrition or supportive feeding viagastric tube [28]. Concerning the potentially serious complications, Re-RT was performed in highly selected group of patients in clinical practice. In our study, 16 patients (53.3%) had grade 1–2 RE, but most of the toxicities were manageable, and only one grade 3 RE was observed. We should pay more attention to these patients, and intravenous nutrition or nasal feeding diet are optional treatment strategies after Re-RT. Myelosuppression was another concern in Re-RT. 43.3% of patients suffered from myelosuppression, which might relate to the concurrent chemotherapy (8/13). Our results showed that grade 1–2 radiation pneumonitis, stenosis of the esophagus,and esophageal fistulas were noted in 1 patient, 2 patients, and 4 patients, respectively, due to the use of IMRT/VMAT. Zhou et al. [23] reported that the esophageal fistulas was observed in 11 cases (20.0%). Chen et al. [20] showed that esophagotracheal fistulas in 5 patients and esophageal perforation in 2 patients were identified. In the current study, radiation pneumonitis occurred in only 3.3%, and esophagotracheal fistulas or esophageal perforation occurred in 13.3%, which were significantly better than previous studies [7, 12, 18]. The late toxic effects in this study were rarely seen. The possible reason was that the outcomes after Re-RT was rather poor even if there were concerning OAR dose limitations.

There were several limitations in our study. On the one hand, due to a single-center retrospective study and the rarity of Re-RT treatment, the number of cases was limited. On the other hand, some data concerning improvement of symptoms such as dysphagia, weight loss, and quality of life score (QoL score) after Re-RT were not available in current study. In daily clinical practice, we should carefully formulate individualized treatment plans and highly select patients suitable for re-RT and nutrition and close follow-up should be investigated in future studies.

## Conclusion

Our results demonstrated that IMRT/VMAT-based Re-RT was an effective therapeutic option for recurrent ESCC patients with local primary-recurrence compared with chemotherapy alone or observation, and toxicities were tolerable. Besides, we found that Re-RT dose and concurrent chemotherapy did not improve the survival time after Re-RT. Therefore, it is necessary to further analyze a larger series of patients in a multi-center setting.

## Electronic supplementary material

Below is the link to the electronic supplementary material.

**Additional File 1: Supplementary Table S1-S4**

**Additional File 2: Fig. S1.** Kaplan-Meier survival curves. (A, D) Survival of patients with PS = score 0–1 versus PS score = 2. (B, E) Survival of patients who received initial RT dose > 60 Gy versus ≤ 60 Gy. (C, F) Survival of patients who had an RFI ≤ 12 months versus RFI > 12 months

**Additional File 3: Fig. S2.** Recurrence-free interval of patients receiving Re-RT dose > 60 Gy versus ≤ 60 Gy

## Data Availability

All data generated or analyzed during this study are included in this article and its supplementary information files.

## Abbreviations

ESCC: Esophageal squamous cell carcinoma
Re-RT: Re-irradiation
IMRT: Intensity modulated radiation therapy
VMAT: Volumetric modulated arc therapy
OS: Overall survival
ARS: After recurrence survival
RFI: Recurrence-free interval
EC: Esophageal cancer
dCRT: Definitive chemoradiotherapy
LR: Locoregional recurrence
REC: Recurrent esophageal cancer
3D-CRT: Three-dimensional conformal radiation
RT: Radiotherapy
Re-CRT: Re-chemoradiotherapy
PF: Cisplatin and fluorouracil
TP: Paclitaxel and cisplatin
CTCAE: National Cancer Institute Common Terminology Criteria for Adverse Events
HRs: Hazard ratios
CIs: Confidence intervals
RE: Radiation esophagitis
OAR: Organ at risk
