# Supplementary material for: Re-irradiation for local primary-recurrence esophageal squamous cell carcinoma treated with IMRT/VMAT
Source: Radiat Oncol. 2023 Jul 10;18:114. doi: 10.1186/s13014-023-02265-w (PMC10334638; doi:10.1186/s13014-023-02265-w)
Supplement: Supplementary file 1 — Additional File 1: Supplementary Table S1-S4 [file 13014_2023_2265_MOESM1_ESM.docx]

**Supplementary**

**Table S1 Characteristics of 30 ESCC patients with locally primary recurrence at re-RT**

| Variable | Number | Percent |
| --- | --- | --- |
| Gender |  |  |
| Male | 24 | 80.0 |
| Female | 6 | 20.0 |
| Age |  |  |
| ≤65 | 10 | 33.3 |
| >65 | 20 | 66.7 |
| PS |  |  |
| 1 | 11 | 36.7 |
| 2 | 19 | 63.3 |
| RFI (months) |  |  |
| ≤12 | 9 | 30.0 |
| >12 | 21 | 70.0 |
| Tumor length at recurrence (cm) |  |  |
| ≤5 | 1 | 3.3 |
| 5-10 | 25 | 83.3 |
| >10 | 4 | 13.3 |
| Re-RT field |  |  |
| In field | 24 | 80.0 |
| Marginal | 6 | 20.0 |
| Concurrent chemotherapy |  |  |
| Yes | 13 | 43.3 |
| No | 17 | 56.7 |
| Re-Radiotherapy technique |  |  |
| IMRT | 10 | 33.3 |
| VMAT | 20 | 66.7 |
| GTV dose (Gy) at initial radiation dose |  |  |
| ≤60 | 13 | 43.3 |
| >60 | 17 | 56.7 |
| PTV dose (Gy) at initial radiation dose |  |  |
| ≤50.4 | 19 | 63.3 |
| >50.4 | 11 | 36.7 |
| GTV dose (Gy) at Re-RT |  |  |
| < 40 | 4 | 13.3 |
| 40-60 | 23 | 76.7 |
| > 60 | 3 | 10.0 |

**Table S2 The doses to OAR at Re-RT**

| Organs | | Median doses |
| --- | --- | --- |
| Left lung | V5 (%) | 49.6 (25.1-75.3) |
|  | V20 (%) | 13.0 (1.2-25.4) |
| Right lung | V5 (%) | 52.7 (24.1-73.1) |
|  | V20 (%) | 10.5 (0.9-21.6) |
| Spinal cord | Dmax (Gy) | 32.9 (0.2-43.0) |
| Heart | Dmean (Gy) | 12.8 (0.2-30.5) |

**Table S3** **Cox model analysis of the OS for 130 local recurrent ESCC patients**

| Variable | Number (%) | OS | | | | ARS | | | |
| --- | --- | --- | --- | --- | --- | --- | --- | --- | --- |
|  |  | Univariate | | Multivariate | | Univariate | | Multivariate | |
|  |  | HR(95%CI) | *P* | HR(95%CI) | *P* | HR(95%CI) | *P* | HR(95%CI) | *P* |
| Age |  | 1.197(0.837-1.712) | 0.324 |  |  | 1.254(0.880-1.785) | 0.210 |  |  |
| ≤65 | 57(43.8) |  |  |  |  |  |  |  |  |
| >65 | 73(56.2) |  |  |  |  |  |  |  |  |
| Gender |  | 1.127(0.760-1.670) | 0.553 |  |  | 1.345(0.906-1.998) | 0.142 |  |  |
| Male | 96(73.8) |  |  |  |  |  |  |  |  |
| Female | 34(26.2) |  |  |  |  |  |  |  |  |
| PS |  | 1.545(1.067-2.236) | **0.021*** | 1.676(1.148-2.449) | **0.008*** | 1.362(0.949-1.954) | **0.094*** | - | - |
| 0/1 | 82(63.1) |  |  |  |  |  |  |  |  |
| 2 | 48(36.9) |  |  |  |  |  |  |  |  |
| T stage |  | 1.345(0.822-2.200) | 0.238 |  |  | 1.010(0.619-1.648) | 0.969 |  |  |
| 1/2 | 19(14.6) |  |  |  |  |  |  |  |  |
| 3/4 | 111(85.4) |  |  |  |  |  |  |  |  |
| N stage |  | 1.347(0.853-2.130) | 0.202 |  |  | 1.168(0.742-1.839) | 0.503 |  |  |
| 0/1 | 107(82.3) |  |  |  |  |  |  |  |  |
| 2/3 | 23(17.7) |  |  |  |  |  |  |  |  |
| Clinical stage |  | 1.067(0.720-1.582) | 0.746 |  |  | 1.032(0.694-1.535) | 0.877 |  |  |
| Ⅰ/Ⅱ | 34(26.2) |  |  |  |  |  |  |  |  |
| Ⅲ/Ⅳ | 96(73.8) |  |  |  |  |  |  |  |  |
| Tumor location |  | 0.936(0.656-1.335) | 0.714 |  |  | 0.965(0.675-1.380) | 0.846 |  |  |
| Neck, upper and middle thoracic | 80(61.5) |  |  |  |  |  |  |  |  |
| Lower thoracic | 50(38.5) |  |  |  |  |  |  |  |  |
| Length(cm) |  | 1.252(0.884-1.774) | 0.206 |  |  | 0.991(0.701-1.400) | 0.957 |  |  |
| ≤5 | 66(50.8) |  |  |  |  |  |  |  |  |
| >5 | 64(49.2) |  |  |  |  |  |  |  |  |
| Radiotherapy technique |  | 1.357(0.936-1.968) | 0.108 |  |  | 0.993(0.691-1.427) | 0.970 |  |  |
| IMRT | 51(39.2) |  |  |  |  |  |  |  |  |
| VMAT | 79(60.8) |  |  |  |  |  |  |  |  |
| Initial radiation dose (Gy) |  | 0.730(0.513-1.039) | **0.080*** | 0.671(0.460-0.980) | **0.039*** | 0.864(0.607-1.230) | 0.417 |  |  |
| ≤60 | 75(57.7) |  |  |  |  |  |  |  |  |
| >60 | 55(42.3) |  |  |  |  |  |  |  |  |
| Concurrence chemotherapy |  | 0.996(0.672-1.475) | 0.983 |  |  | 1.001(0.679-1.474) | 0.998 |  |  |
| Yes | 92(70.8) |  |  |  |  |  |  |  |  |
| No | 38(29.2) |  |  |  |  |  |  |  |  |
| RFI (months) |  | 0.244(0.165-0.361) | **<0.001*** | 0.178(0.111-0.286) | **<0.001*** | 0.755(0.528-1.082) | 0.126 |  |  |
| ≤12 | 80(61.5) |  |  |  |  |  |  |  |  |
| >12 | 50(38.5) |  |  |  |  |  |  |  |  |
| Salvage treatment |  |  |  |  |  |  |  |  |  |
| Without treatment | 58(44.6) | reference |  | reference |  | reference |  |  |  |
| Re-RT±Chemotherapy | 30(23.1) | 0.367(0.231-0.583) | **<0.001*** | 0.600(0.365-0.985) | **0.043*** | 0.647(0.413-1.014) | **0.057*** | **-** | **-** |
| Chemotherapy alone | 29(22.3) | 0.603(0.384-0.945) | **0.027*** | 0.426(0.264-0.689) | **<0.001*** | 0.613(0.391-0.963) | **0.034*** | **-** | **-** |
| Esophageal stents | 8(6.2) | 0.549(0.258-1.167) | 0.119 | 0.294(0.129-0.670) | **0.004*** | 0.478(0.214-1.069) | **0.072*** | - | - |
| Others | 5(3.8) | 0.461(0.184-1.155) | 0.098 | 0.491(0.193-1.244) | 0.134 | 0.561(0.224-1.405) | 0.217 |  |  |

* Univariate analysis was used to calculate the p value of variables, and then multivariate analysis was performed for variables with p < 0.1 to analyze independent risk factors.

**Table S4 Causes of death**

| Cause | N(%) |
| --- | --- |
| Metastasis or uncontrolled disease | 17(56.7) |
| Dysphagia | 6(20.0) |
| Gastrointestinal bleeding | 3(10.0) |
| Esophageal fistula or perforation | 1(3.3) |
| Others | 3(10.0) |
