# Supplementary figures and images for: Re-irradiation for local primary-recurrence esophageal squamous cell carcinoma treated with IMRT/VMAT
Source: Radiat Oncol. 2023 Jul 10;18:114. doi: 10.1186/s13014-023-02265-w (PMC10334638; doi:10.1186/s13014-023-02265-w)

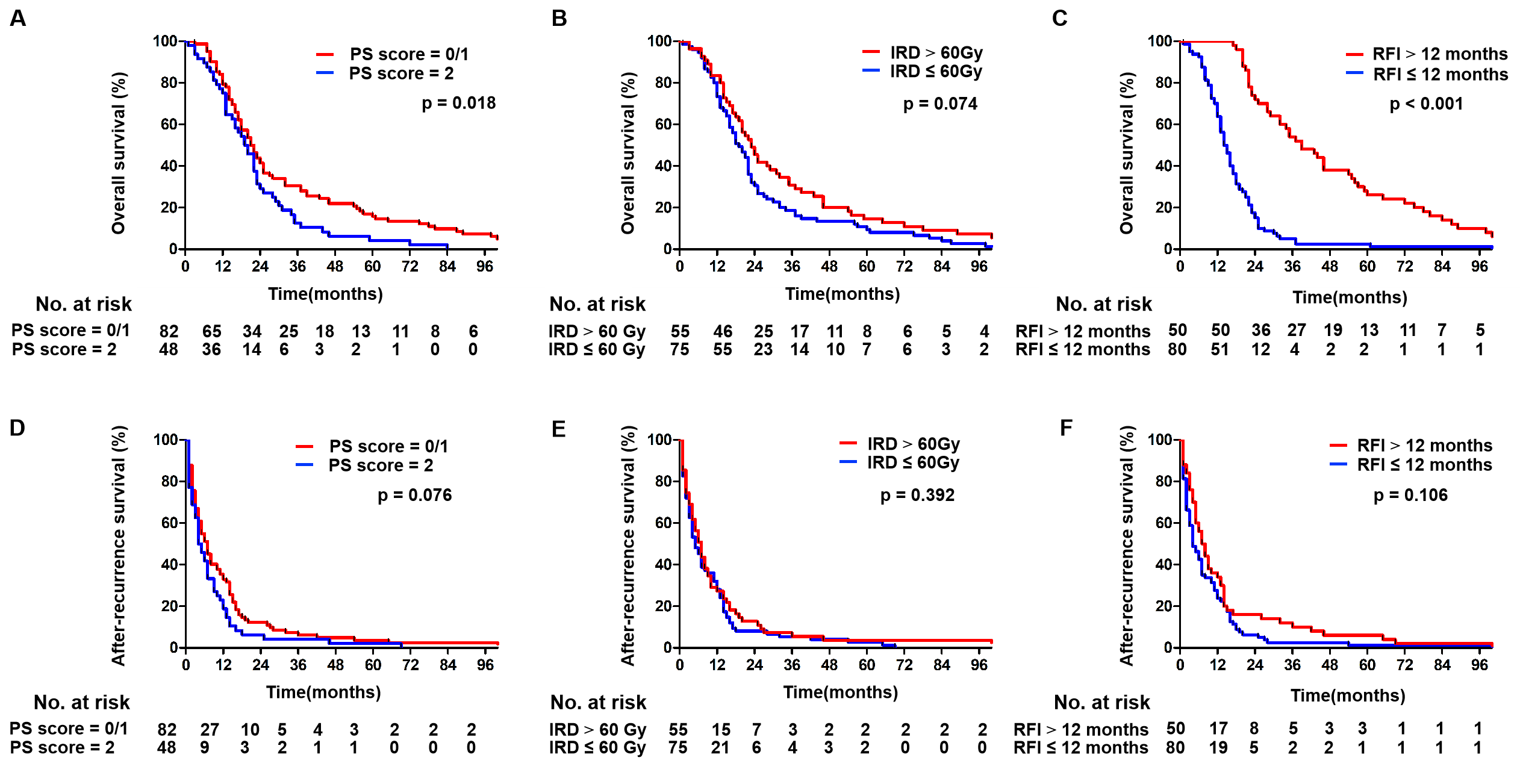

Supplement: Supplementary file 2 — Additional File 2: Fig. S1. Kaplan-Meier survival curves. (A, D) Survival of patients with PS = score 0–1 versus PS score = 2. (B, E) Survival of patients who received initial RT dose > 60 Gy versus ≤ 60 Gy. (C, F) Survival of patients who had an RFI ≤ 12 months versus RFI > 12 months [file 13014_2023_2265_MOESM2_ESM.tif]

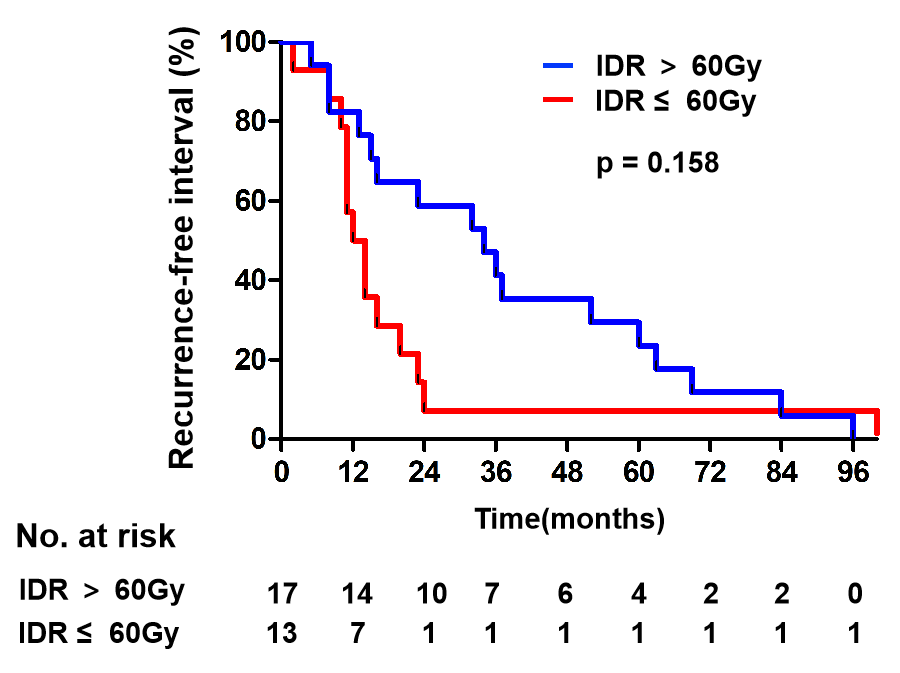

Supplement: Supplementary file 3 — Additional File 3: Fig. S2. Recurrence-free interval of patients receiving Re-RT dose > 60 Gy versus ≤ 60 Gy [file 13014_2023_2265_MOESM3_ESM.tif]
